# From Self-Monitoring to Better Health: Changes in Diet Quality, Body Composition and Cardiometabolic Markers Following Use of the Dietary Diary App in a Single-Arm Study

**DOI:** 10.3390/nu18183003

**Published:** 2026-09-14

**Authors:** Lidia Wadolowska, Ewa Niedzwiedzka, Beata Stasiewicz, Monika Jablonska, Joanna Kowalkowska

**Affiliations:** 1 Department of Human Nutrition, Faculty of Food Science, University of Warmia and Mazury in Olsztyn, Sloneczna 45 F, 10-718 Olsztyn, Poland; lidia.wadolowska@uwm.edu.pl (L.W.); beata.stasiewicz@uwm.edu.pl (B.S.); monika.jablonska@uwm.edu.pl (M.J.); joanna.kowalkowska@uwm.edu.pl (J.K.); 2 Department of Zoology, Faculty of Biology and Biotechnology, University of Warmia and Mazury in Olsztyn, Oczapowskiego 5, 10-718 Olsztyn, Poland

**Keywords:** mobile applications, diet-related diseases, dietary diary, diet quality, nutritional prevention, dietary recommendations, body composition, cardiometabolic disorders

## Abstract

**Background/Objectives:** Despite the rapid development of personalised nutrition, new tools are still needed to support and improve diet quality and prevent diet-related diseases in individuals who do not seek or require personalised dietary advice. The study analysed changes in diet quality, body composition and cardiometabolic markers in two groups of the Dietary Diary app (DDApp) users: (i) after 10 weeks in invited app users, (ii) after one year in voluntary app users who, after 10 weeks, were not encouraged to continue using the app. **Methods:** The app underwent testing with 62 adult participants (aged 18–69 years). App users’ body composition, systolic (SBP) and diastolic (DBP) blood pressure, total cholesterol (TC) and fasting blood glucose in capillary blood (FBG) were measured before (62 users), after 10 weeks (62) and after a year (30). Dietary data were collected, and pro-Healthy Diet Index (pHDI) and non-Healthy Diet Index (nHDI) were calculated. **Results:** After 10 weeks, invited app users recorded decreased nHDI (mean ± standard deviation: from 15.5 ± 6.2 to 12.1 ± 4.9% points; *p* < 0.001), body weight (81.5 ± 18.3 to 79.7 ± 18.0 kg; *p* < 0.001), body mass index (28.6 ± 6.1 to 28.0 ± 6.1 kg/m^2^; *p* < 0.001), waist circumference (91.2 ± 14.8 to 89.7 ± 14.4 cm; *p* = 0.019), fat content (36.6 ± 7.9 to 35.1 ± 8.5% bw.; *p* < 0.001), FBG (108.3 ± 30.1 to 93.5 ± 17.3 mg/dL; *p* < 0.001), TC (199.1 ± 36.6 to 169.3 ± 25.5 mg/dL; *p* < 0.001), SBP (131.3 ± 16.5 to 123.5 ± 15.6 mmHg; *p* < 0.001), DBP (84.8 ± 9.8 to 80.1 ± 9.3 mmHg; *p* < 0.001), along with increased fat-free mass/fat mass ratio (1.9 ± 0.9 to 2.1 ± 0.9; *p* < 0.001) and muscle mass/fat mass ratio (0.9 ± 0.5 to 1.0 ± 0.5; *p* < 0.001). After a year, voluntary app users had decreased nHDI (from 16.7 ± 6.7 to 13.5 ± 6.1% points; *p* = 0.017), visceral fat tissue (1.9 ± 1.8 to 1.8 ± 1.7 kg; *p* = 0.045) and SBP (129.9 ± 15.9 to 125.0 ± 13.5 mmHg; *p* = 0.015). **Conclusions:** DDApp may support dietary behaviour change through self-monitoring, with favourable short-term reductions in nHDI and improvements in body composition and selected cardiometabolic markers. Some benefits persisted after one year.

## 1. Introduction

Chronic non-communicable diseases (NCDs), including cardiovascular diseases, cancer, type 2 diabetes and chronic respiratory diseases, remain one of the main public health challenges in the European Union [1]. Of particular significance is the rapid rise in the prevalence of overweight and obesity, which increases the risk of glucose and lipid metabolism disorders, hypertension and cardiovascular diseases, creating a complex network of interrelated health conditions [1,2].

Reducing the burden of NCDs requires lifestyle modifications and the reduction of major modifiable risk factors, including excessive alcohol consumption, smoking, poor diet and low levels of physical activity. National and international dietary prevention guidelines set out the direction of dietary changes [3,4]. Despite the widespread availability of these recommendations and numerous efforts to disseminate them, many individuals have difficulty implementing and maintaining recommended dietary behaviours [5]. The most commonly reported barriers to adhering to dietary recommendations include lack of time, declining motivation, food cost, insufficient cooking skills and difficulty making lasting changes to ingrained eating habits [5,6].

These barriers highlight the need for approaches that not only provide dietary recommendations but also facilitate their implementation and maintenance in everyday life [5,6]. Alongside the rapid development of personalised nutrition, simple population-oriented approaches remain relevant, particularly for individuals who do not seek or require individualised dietary counselling [7,8]. Such approaches may help translate general dietary recommendations into everyday behaviours and support dietary self-management [2,9,10,11,12,13].

In this context, tools that support self-monitoring of dietary habits and the achievement of dietary goals are becoming increasingly important. The traditional paper-based food diary is a useful tool for collecting dietary data (food record), but keeping a running record of food intake can be time-consuming and discourage many users from maintaining a regular routine [4]. This is particularly true for people who want to monitor their diet to improve it. The development of mobile technology has enabled the transfer of self-monitoring to mobile health (mHealth) applications. According to the World Health Organization (WHO), mHealth refers to medical practices and public health activities supported by mobile devices and related technologies [9]. Mobile applications can facilitate dietary recording, goal setting and progress monitoring, thereby supporting adherence to dietary recommendations and the achievement of individual health-related goals [2,10,11].

Most available mHealth tools aimed at improving diet require a relatively high level of user engagement and detailed recording of the type, quantity and portion size of food consumed, and the results obtained mainly relate to the energy and nutritional value of the diet [12,13,14]. This approach may be time-consuming and, in practice, requires the regular entry of large amounts of data. In mobile applications, the complexity of the recording process and the poor alignment of features with users’ everyday needs are significant barriers to their adoption. Conversely, ease of use, simple recording, clear functions and useful feedback can encourage regular app use [12]. Maintaining user engagement is one of the most frequently reported challenges in studies examining the effectiveness of mHealth in improving dietary habits [15]. Furthermore, the reliability of dietary information generated by such applications depends, among other things, on correctly identifying the food consumed, accurately assessing portion sizes and validating the tool used [16,17,18]. Therefore, results obtained from non-validated apps cannot be assumed to accurately reflect actual energy and nutrient intake.

The Dietary Diary App (DDApp) was developed as a population-oriented alternative to tools requiring a comprehensive food diary. It is a simple self-monitoring tool that does not require detailed recording of all foods consumed or calculation of energy and nutrient intake, but focuses on selected food groups of key health importance and indicates the desired direction for changes in their intake, thereby supporting users in following population-based dietary recommendations [19,20]. The DDApp has previously been described as a tool supporting simplified self-monitoring of dietary patterns and the achievement of nutritional goals based on selected food groups [19,20].

The aim of this single-arm study was to assess changes in diet quality, body composition and cardiometabolic markers in two groups of the Dietary Diary app users: (i) after 10 weeks in invited app users and (ii) after one year in voluntary app users who had completed the initial 10-week protocol and were subsequently not encouraged to continue using the app. We hypothesised that favourable changes in diet quality and selected health markers would be observed after 10 weeks and that some of these changes might persist at the one-year follow-up despite less frequent voluntary app use.

## 2. Materials and Methods

### 2.1. Dietary Diary App and Its Concept

The DDApp is intended for the general adult population and those individuals who do not seek or require personalised dietary advice. The app includes clearly defined nutritional goals developed from population-based nutritional prevention recommendations and scientific evidence on the relationship between diet and health [3,4,21,22]. These goals consider both national and international recommendations for nutritional prevention aimed at reducing diet-related diseases. Additionally, the recommended diet emphasises a higher intake of plant-based foods and a lower intake of highly processed foods, taking into account their environmental impact [3,4,21,22,23,24,25].

The DDApp is an mHealth intervention focused on self-management and providing a simple guide to desired changes in the consumption of key health-related food groups.

The DDApp forgoes detailed recording of food intake and calculating the energy and nutritional value of the diet, focusing on a few selected food groups with documented impact on health [19]. Based on the collected evidence, nutritional goals were defined as the minimum or maximum number of servings to be consumed.

The main panel of the DDApp includes 11 food items, each with defined nutritional goals. A recommended minimum number of servings (per day or week) was determined for six pro-healthy foods, whereas a maximum number of servings (per week) was determined for five not-so-healthy foods. The following nutritional goals were established based on national and international population-based dietary recommendations: vegetables ≥ 3 servings/day, dairy foods ≥ 2 servings/day, fruits ≥ 2 servings/day, whole grains ≥ 1 serving/day, fish/seafood ≥ 2 servings/week, legumes/nuts/seeds ≥ 1 serving/week, meats ≤ 5 servings/week, sweets: ≤1 serving/week, sweetened/energy drinks: ≤1 serving/week, fast foods: ≤1 serving/week, alcoholic beverages: ≤1 serving/week.

Using the application is very simple and does not take much time (2–3 min a day): the user records every serving of 11 food items included in the app (just a “click”). The app is available in Polish, free of charge, at the Google Play Store and the Apple App Store, or via the DietTools4U website [26].

In this article, we use the food group names proposed by McManus of Harvard University [27]. Foods that have a potentially beneficial effect on health are called “good for health”, while foods that may have a negative effect on health are called “not-so-good for health”.

### 2.2. Ethical Approval

The Bioethics Committee of the Faculty of Medical Sciences, University of Warmia and Mazury in Olsztyn approved the study on 21 January 2021 (Resolution No. 2/2021) as an annex to ethical approval obtained on 17 June 2010 (Resolution No. 20/2010). The study was performed in accordance with relevant guidelines and regulations, including the Declaration of Helsinki for Medical Research Involving Human Subjects. Written informed consent was obtained from all study participants. Participants were instructed to report any suspected adverse events. No adverse events were reported.

### 2.3. Study Design and Participants

**Figure 1:**
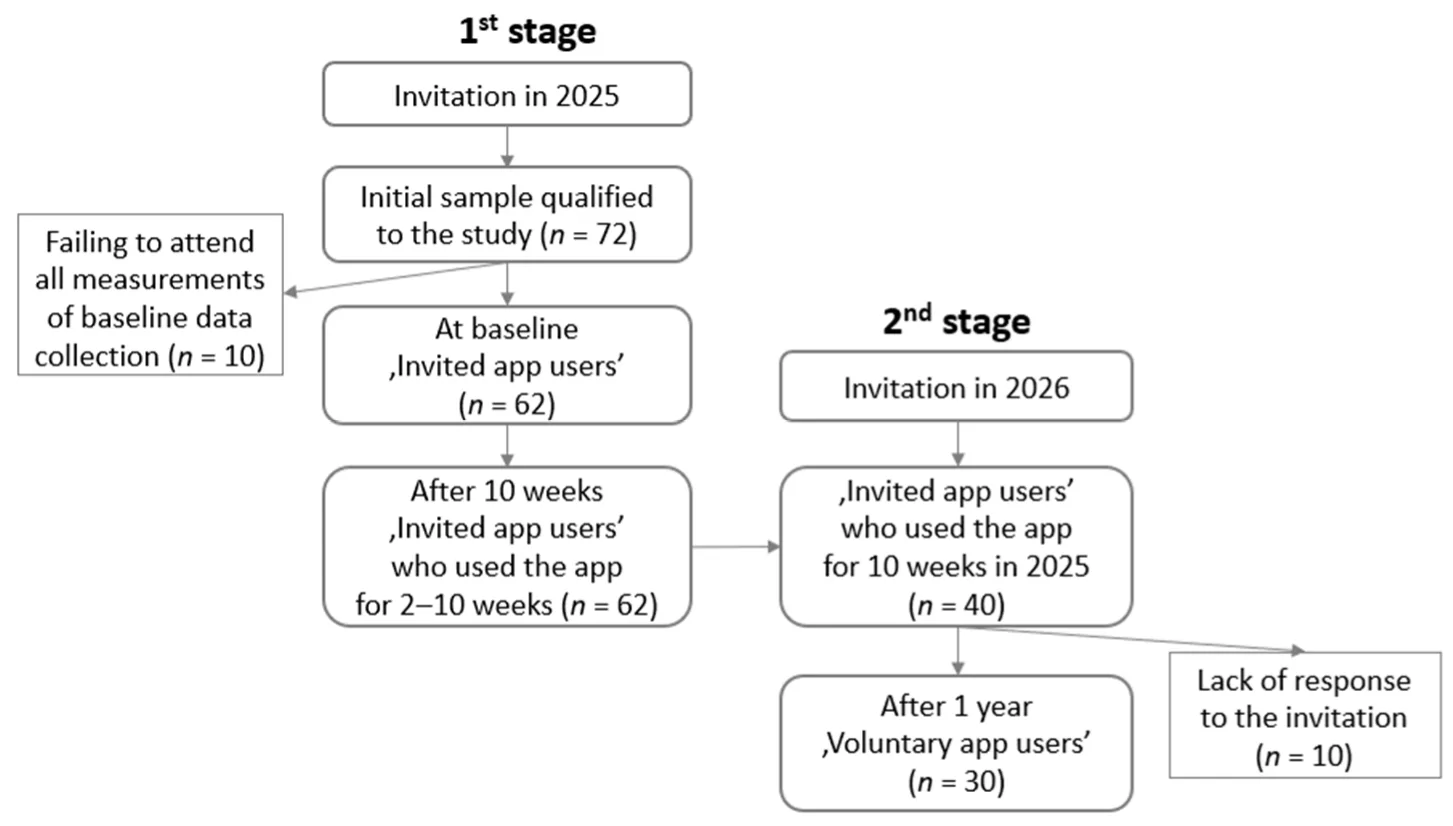
Flowchart of sample collection.

**Figure 2:**
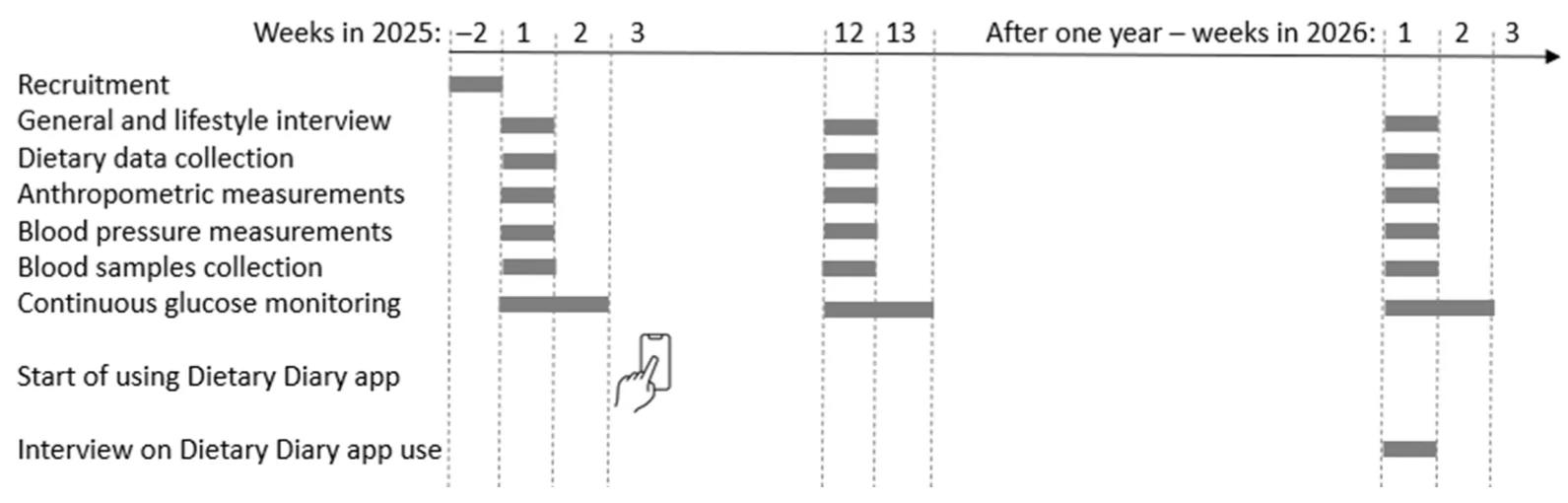
Study design and data collection.

The study was divided into two stages according to two aims. Data were collected three times: at baseline, after 10 weeks and after a year, each time during two visits to the study centre and one online data collection (Figure 1 and Figure 2).

To analyse the data collected at the 1st stage, we used the intention-to-use (ITU) approach. The intention-to-treat (ITT) approach is fundamental in clinical trials [28]. It reflects real-world conditions and yields more conservative interpretations because some participants do not strictly adhere to the study protocol (e.g., medication or diet regimens). On the other hand, the per-protocol (PP) approach includes only results from adherent participants, reflecting an ideal-world scenario and supporting stronger interpretation.

For the 1st stage (in 2025), recruitment was conducted through advertisements and personal contacts. The inclusion criteria were age 18–70 years; healthy or unhealthy subjects who did not require a therapeutic diet conflicting with the nutritional goals outlined in the DDApp; past use of mHealth tools until the recruitment to the study began; informed consent to participate in the study; willingness to provide cardiometabolic markers and use of the data. For example, subjects were classified as unhealthy if they declared hypertension and were taking (or not) blood pressure-lowering drugs; declared prediabetes or diabetes and were taking (or not) glucose-lowering drugs; were on a diet prescribed by a doctor for dyslipidaemia or diabetes; were on a veggie, high-protein or lactose-free diet. The exclusion criteria were age <18 or >70 years; pregnancy or breastfeeding; current use of individual dietary counselling; current participation in other nutritional programs; current use of other mHealth tools; diseases requiring currently specialised dietary interventions; current use of diet conflicting with the nutritional goals outlined in the DDApp or very restrictive diets (e.g., fruitarianism, very low calorie diet); planned changes in medication dosage throughout the study period (10 weeks); bariatric surgery in the past; a cognitive impairment that would not allow for understanding and responding to questions that were asked by the interviewer.

For the 1st stage of the study, 72 subjects were qualified. Due to missing baseline measurements, 10 subjects were excluded. After baseline data collection, participants were encouraged to use the app daily for 10 weeks and achieve their nutritional goals. At baseline and after 10 weeks, 62 adults aged 18–69 years participated in the study. The sample also included subjects with chronic diseases who met the inclusion and exclusion criteria (Table 1). Of the 62 participants, 40 adhered to the study protocol and used the app daily throughout the entire 10-week intervention period. The remaining 22 participants started using the app but used it for 2–9 weeks. Importantly, these 22 participants still completed both the baseline and 10-week follow-up measurements and were therefore retained in the ITU analysis.

For the 2nd stage (in 2026), invitations to participate were restricted to those who had strictly adhered to the study protocol during the 1st stage by using the app daily for the full 10-week intervention period. All participants (*n* = 40) who used the app for 10 weeks in 2025 were invited, but after the 1st stage, they were not encouraged to continue using the app or other mHealth apps, nor were they provided with written/oral dietary recommendations. Thirty participants took part in the 2nd stage of the study.

Participants from the 1st stage of the study were labelled as invited app users regardless of the duration of app use (2–9 or 10 weeks), while participants from the 2nd stage were labelled as voluntary app users.

### 2.4. Dietary, Lifestyle and Socioeconomic Data

Dietary data were collected using the food frequency method with an online version of the KomPAN^®^ questionnaire covering the last 12 months [29,30]. The KomPAN^®^ questionnaire (online version), containing closed questions, was also used to collect lifestyle and socioeconomic data [31]. Data on the prevalence of chronic diseases and medication taking were gathered through an open-ended interview. Data on the frequency of app use during the year were collected (a question with four answer choices).

Data regarding the consumption frequency of 24 food groups were obtained. The respondents could choose one of six categories (next converted into daily frequency): never (0 times/day), 1–3 times a month (0.06 times/day), once a week (0.14 times/day), a few times a week (0.5 times/day), once a day (1.0 time/day), or a few times a day (2.0 times/day), in accordance with the KomPAN^®^ manual guide [29].

Diet quality was assessed using two dietary scores: the pro-Healthy Diet Index (pHDI) and non-Healthy Diet Index (nHDI) [29]. Both dietary scores were calculated in accordance with the questionnaire manual guide and expressed as percentage points (range 0–100) [29]. Higher pHDI scores resulted from a greater frequency of consumption of 10 food groups with a potentially beneficial effect on health (wholemeal bread, coarse-ground groats, milk, fermented milk drinks, fresh cheese curd products, white meat, fish, pulse-based foods, vegetables, fruit), and higher nHDI scores were associated with a greater frequency of consumption of 14 food groups with a potentially adverse effect on health (white bread, white rice/white pasta/fine-ground groats, fried foods, lard, butter, cheese, cured meat/smoked sausages/hot-dogs, red meat, canned meat, sweets, sweetened drinks, energy drinks, alcoholic beverages). Higher pHDI scores were interpreted as better diet quality, while higher nHDI scores were interpreted as worse diet quality [29].

### 2.5. Body Composition and Cardiometabolic Markers

Measurements of body composition, including general body fat content, visceral fat mass, fat-free mass, skeletal muscle mass, weight, height and waist circumference (WC) were obtained according to the International Society for Advancement of Kinanthropometry International Standards for Anthropometric Assessment guidelines (ISAK) [32]. Professional equipment and a measuring tape were used: for measuring height—a portable stadiometer SECA 220, for weight—an electronic digital scale SECA 799, for WC—stretch-resistant tape SECA 201 and for body fat and skeletal muscle mass (using bioelectrical impedance technique)—a SECA medical Body Composition Analyzer (mBCA) 515. Adiposity was determined based on several anthropometric indices: overweight (body mass index, BMI = 25–29.9 kg/m^2^), central obesity (two indices: waist-to-height ratio, WHtR ≥ 0.5 and WC ≥ 80 cm [female] or ≥94 cm [male]) and obesity (two indices: BMI ≥ 30 kg/m^2^ and body fat ≥ 30% bw. [female] or ≥25% bw. [male]) [33,34,35].

Measurements of systolic (SBP) and diastolic (DBP) blood pressure were obtained with Omron M3 Intellisense Automatic Blood Monitor (Omron Healthcare Co., Ltd., Muko, Kyoto, Japan) [36]. Total cholesterol concentration (TC) and fasting blood glucose (FBG) in capillary blood were measured with Accutrend^®^ Plus system (Roche Diagnostics, GmbH, Mannheim, Germany). The tests were performed in the morning, and participants were asked to refrain from eating or drinking (except water) for 8–10 h before the appointment. Fourteen-day continuous glucose monitoring sessions were conducted, using a Freestyle Libre 2 sensor (Abbott Diabetes Care Ltd., Witney, UK,) to determine mean daily continuous glucose (mg/dL) and time in the target glucose range, both measured in interstitial tissue. The target glucose range was 70–180 mg/dL [37]. Cardiometabolic disorders were recorded if the following markers were elevated: FBG ≥ 100 mg/dL, SBP ≥ 140 or DBP ≥ 90 mmHg [38,39,40].

### 2.6. Statistical Analysis

Sample size calculation was based on the expected mean differences between two paired measurements and the standard deviation of the differences [41]. Before the study, it was calculated that a sample size of 34 (number of pairs) is required to achieve a power of 80% and a level of significance of 5% (two-sided), for detecting a mean of the differences of 5 units between pairs, assuming a standard deviation of the differences of 10 units. Furthermore, a post hoc analysis was conducted for means of differences and the standard deviation of the differences achieved in this study; thus, a minimum sample size for pHDI, nHDI, BMI, percentage of fat mass, FBG, TC, SBP and DBP was 25, 30, 11, 11, 25, 17, 21 and 24 (number of pairs), respectively.

Data were presented as means and standard deviations (SDs) for continuous variables and percentages for categorical variables. Absolute difference (AD) and relative difference (RD) between two paired measurements were calculated with mean values as follows: AD = after − baseline, and RD = [(after − baseline) × 100/baseline]. The differences between paired measurements were tested using the non-parametric Wilcoxon test for continuous variables and the McNemar or McNemar–Bowker test for categorical variables. We previously verified the normality of the variables using the Kolmogorov–Smirnov test.

For invited app users, baseline data were compared with data after 10 weeks in 62 participants, regardless of whether they followed the study protocol and used the app daily (*n* = 40) or did not follow the protocol (i.e., did not use the app daily) (*n* = 22). The database was complete for dietary and body composition data, but contained single missing values for cardiometabolic markers, except for TC, which had a higher level of missing data (>25%). In accordance with the general principles of processing continuous data in the intention-to-use approach, and to avoid over-interpretation of results, missing data were supplemented with variable means calculated separately for females and males [28]. To avoid misclassification of elevated TC, percentages for this variable are not presented. The statistical analysis was performed using STATISTICA software (version 13.3 PL; TIBCO Software Inc., Tulsa, USA; StatSoft Polska, Cracow, Poland) and PS IMAGO PRO 10.0, including IBM SPSS Statistics 29 (Predictive Solutions, Cracow, Poland). A *p*-value < 0.05 was considered to be statistically significant.

## 3. Results

### 3.1. Sample Characteristics and App Use

In all subgroups (at baseline, after 10 weeks and after one year), approximately 80% of participants were female, approximately 95% had a higher education and 73–80% were urban residents (Table 1). Fewer than 50% of participants reported a chronic disease or medication use, while 68–74% reported not using a therapeutic diet. None of the unhealthy participants changed their medication regimen during the intervention. Table S1 presents characteristics of the study sample that followed the study protocol for 10 weeks and participated in the study after a year.

Sample characteristics did not change significantly after 10 weeks or after one year, except for age and smoking. In subsequent phases of the study, participants were significantly older. After one year, some voluntary app users had stopped smoking; current smoking decreased from 27% to 10% of the sample (*p* = 0.025).

**Figure 3:**
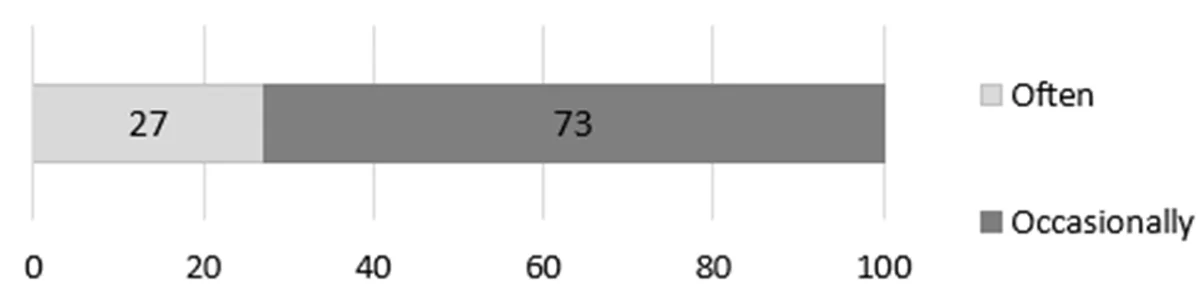
Voluntary app users: App use for a year (% of the sample).

After a year, 27% of voluntary app users reported frequent app use, while 73% of them used the app occasionally (Figure 3). No one reported daily use or non-use of the app. Table S2 presents the results of an assessment of the app usability by voluntary users after a year.

### 3.2. Diet Quality Changes in Invited App Users

After 10 weeks, the nHDI significantly decreased on average by −21.9% of the initial value (from 15.5 to 12.1%points; *p* < 0.001; Table 2). The change was a result of a decrease in frequency of consumption of six food items: white bread by −45.0% of initial value (*p* < 0.001), white rice/white pasta/fine-ground groats by −27.3% of initial value (*p* = 0.004), butter by −24.1% of the initial value (*p* = 0.047), sweets by −21.2% of the initial value (*p* = 0.035), cured meat/smoked sausages/hot-dogs by −20.8% of the initial value (*p* = 0.021) and fried foods by −20.0% of the initial value (*p* = 0.042). No significant change in pHDI was found, although the frequency of consumption of two food items significantly increased: fermented milk drinks by 21.1% of the initial value (*p* = 0.023) and vegetables by 18.0% of the initial value (*p* = 0.004).

### 3.3. Diet Quality Changes in Voluntary App Users

After 10 weeks, the nHDI significantly decreased by an average of 24.0% of the initial value (from 16.7 to 12.7% points; *p* = 0.006; Table 3). The change was a result of a decrease in frequency of consumption of four food items: white bread by −41.8% of initial value (*p* = 0.022), white rice/white pasta/fine-ground groats by −34.3% of initial value (*p* = 0.019), tinned meats by −33.3 of initial value (*p* = 0.050), sweets by −32.1% of the initial value (*p* = 0.022). No significant change in pHDI was found.

After a year, the nHDI significantly decreased on average by −19.2% of the initial value (from 16.7 to 13.5%points; *p* = 0.017). It was a result of a decrease in frequency of consumption of 4 food items: tinned meat by −66.7% of the initial value (*p* = 0.033), white bread by −31.9% of initial value (*p* = 0.046), white rice/white pasta/fine-ground groats by −28.6% of the initial value (*p* = 0.049), cured meat/smoked sausages/hot-dogs by −27.9% of the initial value (*p* = 0.020).

### 3.4. Body Mass and Composition Changes in Invited App Users

After 10 weeks, a significant decrease in six markers was found, on average: fat mass by −6.2% of the initial value (−1.9 kg; *p* < 0.001), fat content by −4.1% of the initial value (−1.5%bw.; *p* < 0.001), body weight by −2.2% of the initial value (−1.8 kg; *p* < 0.001), BMI by −2.1% of the initial value (−0.6 kg/m^2^; *p* < 0.001), WHtR by −1.9% of the initial value (−0.01; *p* = 0.022) and waist circumference by −1.6% of the initial value (−1.5 cm; *p* = 0.019) (Table 4). In contrast, a significant increase in two ratios: muscle mass/fat mass ratio by 11.1% of the initial value (*p* < 0.001) and fat-free mass/fat mass ratio by 10.5% of the initial value (*p* < 0.001) was observed. An insignificant decrease in prevalence of high BMI (≥30 kg/m^2^; *p* = 0.500), high waist circumference (*p* = 0.344) and high body fat (*p* = 0.125) by −7 to −13% of the initial prevalence was recorded (Table 5).

### 3.5. Body Mass and Composition Changes in Voluntary App Users

After 10 weeks, a significant decrease in four markers was found, on average: fat mass by −7.7% of the initial value (−2.2 kg; *p* < 0.001), fat content by −5.3% of the initial value (−1.9%bw.; *p* < 0.001), BMI by −2.5% of the initial value (−0.7 kg/m^2^; *p* < 0.001), body weight by −2.4% of the initial value (−1.9 kg; *p* < 0.001) (Table 6). In contrast, a significant increase in two ratios: muscle mass/fat mass ratio by 11.1% of the initial value (*p* < 0.001) and fat-free mass/fat mass ratio by 10.5% of the initial value (*p* < 0.001) was observed.

After one year, visceral fat tissue decreased significantly by 5.3% of the initial value (−0.1 kg; *p* = 0.045) (Table 6). No significant differences were found in prevalence of body composition disorders after 10 weeks and after one year (Table 7).

### 3.6. Cardiometabolic Marker Changes in Invited App Users

After 10 weeks, a significant decrease in four markers was found, on average: TC by −15.0% of the initial value (from 199.1 to 169.3 mg/dL; *p* < 0.001), FBG by −13.7% of the initial value (from 108.3 to 93.5 mg/dL; *p* < 0.001), SBP by −5.9% of the initial value (from 131.3 to 123.5 mmHg; *p* < 0.001), and DBP by −5.5% of the initial value (from 84.8 to 80.1 mmHg; *p* < 0.001) (Table 4). A significant decrease in elevated SBP or DBP by −62% of the initial prevalence (from 42 to 16% of the sample; *p* < 0.001), and elevated FBG by −41% of the initial prevalence (from 58 to 34% of the sample; *p* = 0.001) was found (Table 5).

### 3.7. Cardiometabolic Marker Changes in Voluntary App Users

After 10 weeks, a significant decrease in four markers was found, on average: FBG by −17.9% of the initial value (from 111.3 to 91.4 mg/dL; *p* < 0.001), TC by −16.1% of the initial value (from 201.1 to 168.7 mg/dL; *p* < 0.001), SBP by −7.3% of the initial value (from 129.9 to 120.4 mmHg; *p* < 0.001) and DBP by −6.9% of the initial value (from 84.0 to 78.2 mmHg; *p* < 0.001) (Table 6). The prevalence of elevated SBP or DBP significantly decreased by −70% of the initial prevalence (from 33 to 10% of the sample; *p* = 0.016), and the prevalence of elevated FBG by −47% of the initial prevalence (from 57 to 30% of the sample; *p* = 0.021) (Table 7).

After one year, a significant decrease in SBP by −3.8% of the initial value (from 129.9 to 125.0 mmHg; *p* = 0.015) was found. No significant differences in the prevalence of cardiometabolic disorders were observed after one year (Table 7).

## 4. Discussion

In this study, DDApp users demonstrated favourable within-participant changes in diet quality, body composition, and selected cardiometabolic markers. The most consistent dietary finding was a reduction in nHDI, indicating lower consumption of less healthy foods. The largest changes were observed after 10 weeks, when participants were encouraged to use the app daily, whereas only some outcomes remained significantly improved at the one-year follow-up, namely nHDI, visceral fat tissue and systolic blood pressure. Overall, these findings were broadly consistent with our hypothesis that favourable changes would emerge during the initial intervention period and that some, but not all, would persist despite less frequent voluntary app use.

The study results indicate that the most pronounced and sustained change concerned diet quality, reflected in a significant reduction in the non-Healthy Diet Index. After 10 weeks, the nHDI score was significantly lower than at the start of the study and remained significantly lower even after one year. This change was primarily associated with reduced consumption of highly processed foods rich in saturated fatty acids and sugars. This finding may be significant for dietary prevention, as frequent consumption of less healthy foods is a major factor contributing to obesity and its metabolic complications [42,43]. The direction of the observed changes is consistent with findings from systematic reviews and meta-analyses, suggesting that mHealth interventions can lead to small-to-moderate improvements in dietary patterns. The magnitude and durability of these changes may depend on the nature of the intervention, the type of behaviour being monitored, the study population and the level of user engagement [2,44,45].

However, no significant increase in the pro-Healthy Diet Index (pHDI) was observed, despite increased consumption frequency of two important food groups (fermented dairy products and vegetables). The contrasting trends in the nHDI (a significant decrease) and the pHDI (a non-significant increase) may suggest that, in a short-term self-monitoring-based intervention, it was easier to reduce consumption of less healthy foods than to increase consumption of many health-promoting foods. One possible explanation is that increasing the consumption of health-promoting foods may require not only a change in individual dietary choices, but also, among other things, planning shopping trips, modifying meal patterns and replacing ingrained habits. In this context, self-monitoring, setting specific goals and feedback may be important. These are among the most commonly used behaviour change techniques in mHealth interventions and may support both the implementation of recommendations and the maintenance of user engagement [8,46].

The potential role of self-monitoring is also relevant when considering the persistence of dietary changes over time. Our study indicates that the greatest changes in dietary quality occurred after 10 weeks of using the DDApp; after one year, dietary changes were slightly smaller, but most remained significant. It is important to emphasise that after a year, maintaining the favourable changes in dietary quality resulted from spontaneous, albeit not very regular, use of the app. Therefore, it can be assumed that users who used the app daily for 10 weeks developed healthy eating habits during that time and maintained them throughout the year by using the app occasionally (73%) or often (27%). Thus, the persistence of a lower nHDI coincided with less frequent voluntary app use. The present design does not allow us to determine whether occasional continued use of the DDApp contributed to maintaining these dietary changes. One possible interpretation is that the app may have served as a periodic reminder of previously established dietary goals or as a tool for self-assessment. In the first stage, most participants (65%) used the app daily. Other studies have shown that changes observed during mobile app-based interventions are usually most pronounced in the initial period of use, while the long-term maintenance of dietary changes and regular app use remains a challenge [44,47]. Curtin et al. [44] found that beneficial changes in dietary patterns were most frequently observed up to around the 12th week of the intervention, whereas data on long-term changes and the maintenance of these changes were less clear-cut. Similarly, Salas-Groves et al. [47] noted that, over time, some studies observed declines in user engagement and app use, which may have limited the maintenance of the changes achieved. In this context, the persistence of a lower nHDI at one year is noteworthy, although the selected nature of the one-year subgroup should be considered when comparing these findings with previous studies [44,47].

Beyond diet quality, favourable short-term changes were also observed in body weight and body composition. Among DDApp users, body weight, waist circumference and fat mass decreased. At the same time, markers expressing the ratio of muscle mass or fat-free mass to body fat increased. Taken together, all changes indicate a favourable reduction in body fat while maintaining fat-free body mass. The range of these changes was small, but their direction aligned with findings from systematic reviews and meta-analyses, which generally associated mobile app use with a moderate reduction in body weight, BMI, waist circumference and fat mass [48,49,50,51,52]. Although not all favourable changes in body weight and composition were sustained among DDApp users after one year, visceral fat was significantly lower. This may indicate that certain beneficial changes in fat distribution are sustained, which should be considered particularly beneficial given the pro-inflammatory nature of visceral adipose tissue [33]. Similar observations have been reported in other studies on mobile app-based interventions. These studies have shown that changes in body weight and composition are generally modest, and that maintaining these changes after 6 or 12 months is less certain than those observed over a shorter period [48,50,53].

A similar favourable short-term pattern was observed for selected cardiometabolic markers. After 10 weeks of using the DDApp, both subgroups (62 and 30 participants) showed reductions in fasting glucose and total cholesterol, as well as decreases in systolic and diastolic blood pressure. After one year, the beneficial changes in systolic blood pressure were maintained, while the same tendency was observed for the other markers. These changes are consistent with systematic reviews and meta-analyses indicating that mHealth interventions combining self-monitoring with lifestyle modification may be associated with slight improvements in selected cardiometabolic indicators, although effects are not uniform across all markers [51,54,55]. In the present study, after 10 weeks, the range of the reductions in fasting glucose (by 15–20 mg/dL) and total cholesterol (by 30–32 mg/dL) was greater than the changes reported in other studies, in which improvements in glycaemic and lipid parameters and blood pressure were usually modest, limited to selected markers or insignificant [51,54,56]. Reductions in SBP or DBP (by 5–8 mmHg) were reflected in a reduction in the prevalence of high blood pressure (by 23–26% of the sample), documenting a clinical effect of improved health after 10 weeks.

In contrast to fasting capillary blood glucose, mean daily interstitial glucose and time in the target glucose range assessed by continuous glucose monitoring (CGM) showed no significant changes. These measures reflect different aspects of glucose regulation and should not be considered interchangeable. In individuals without diabetes, mean daily glucose levels are generally relatively low and time spent within conventional glycaemic target ranges is often already high. In the present study, time in the 70–180 mg/dL target range was already 96–97% at baseline, leaving limited room for further improvement. Previous studies in people without diabetes have reported mean 24 h glucose concentrations close to 100 mg/dL and glucose values within the 70–140 mg/dL range for most of the day [57]. Furthermore, a recent systematic review and meta-analysis showed that associations between CGM-derived glycaemic variability and cardiometabolic risk markers in individuals without diabetes remain inconsistent, supporting the need for further prospective research [58]. Therefore, the absence of changes in CGM-derived outcomes in the present study should not be considered inconsistent with the reduction observed in fasting glucose, but indicates that the decrease in fasting glucose was not accompanied by a detectable change in overall daily interstitial glucose exposure. Taken together, the changes observed after 10 weeks were generally in a favourable cardiometabolic direction, whereas their persistence at the one-year follow-up was limited to selected outcomes. The reduction in nHDI occurred concurrently with changes in body composition and cardiometabolic markers and may represent one of several potential contributors to the observed pattern; however, the present study cannot establish whether dietary changes mediated these health-related outcomes. In this context, the self-monitoring function of the DDApp may be particularly relevant. Self-monitoring, goal setting and feedback are widely used behaviour-change techniques in mHealth interventions and may support the implementation of dietary recommendations and maintenance of behavioural changes [8,46].

The simplified structure of the DDApp, which does not require detailed recording of all foods consumed or calculation of energy and nutrient intake, may reduce the burden associated with dietary self-monitoring. Ease of use, limited recording time, clarity of functions and useful feedback have been identified as important facilitators of continued engagement with dietary mobile applications [12]. In the present study, the mean SUS score of 80.3 indicated favourable perceived usability of the DDApp and was within the range previously interpreted as corresponding to good-to-excellent usability [59,60]. This finding supports the practical feasibility of the simplified self-monitoring approach, although perceived usability itself should not be interpreted as evidence of clinical effectiveness.

The influence of other factors on the observed cardiometabolic changes cannot be ruled out. It was shown that some participants stopped smoking after 10 weeks (4–13%) and after one year (17%). Therefore, the observed cardiometabolic changes may also have been influenced by smoking cessation, as smoking is a strong risk factor for cardiovascular and other chronic non-communicable diseases [1]. On the other hand, smoking cessation, while beneficial for health, may be accompanied by increased appetite, higher energy intake and post-cessation weight gain, which could potentially influence dietary behaviours and attenuate improvements in some dietary parameters [61]. However, this mechanism was not directly assessed in the present study and therefore remains speculative. The influence of other factors appears less important. The absence of reported changes to the pharmacotherapy regimen reduces the likelihood of side effects from dosage adjustments, but does not rule out differences in medication-taking regularity. Similarly, no significant changes were found in the reported categories of recreational physical activity during follow-up. However, the self-reported categorical assessment used in this study may not have been sensitive enough to detect smaller reductions in activity volume or intensity. Such changes, if present, could have attenuated the magnitude of the observed metabolic improvements. Consequently, concurrent lifestyle changes and other unmeasured factors may have contributed to the observed outcomes.

### Strengths and Limitations

The study’s strength is its use of an intention-to-use approach based on the intention-to-treat approach, which is fundamental in clinical trials [28]. Statistical analysis at 10 weeks was performed for all participants with baseline and 10-week follow-up data (*n* = 62), including those who used the app for 2–9 weeks (*n* = 22). This approach better reflects the real world, as many people do not follow all the recommendations given by their dietitian or doctor [28]. Therefore, in the current study, the identified effect of app use is attenuated. Previously, we reported changes in diet quality, body composition and cardiometabolic markers in participants who followed the study protocol (PP approach) and used the app daily for 10 weeks (*n* = 40) [20]. The current outcomes (ITU approach) align with those previously described in the PP approach. Considering both approaches, clear, strong conclusions can be drawn showing two possible pro-healthy effects of app use—greater in daily app users and weaker, but also clinically important, in app users in general, regardless of the regularity of app use.

The number of participants (62 or 30) is the second strength of the study. An a priori sample-size calculation was performed before the study, and the number of participants available for the 10-week analysis exceeded the initially estimated requirement. The calculated minimum sample size (power of 80% and a level of significance of 5%) ranged from 11 to 30 paired measurements, depending on the marker. In the present study, the number of pairs needed to detect differences, if any, was two to six times greater for interpreting changes after 10 weeks and adequate for interpreting changes after one year. Despite the adequate number of participants in the second stage of the study (30), their selection method may have introduced selection bias. It is hard to disagree with this, but the second stage was designed to assess outcomes of spontaneous and long-term app use, and the research design fulfilled this role. Furthermore, this design more closely reflects real-life conditions and allows for a more realistic interpretation, which is important in the first study among app users.

The descriptive qualitative food frequency questionnaire (KomPAN^®^) provides a validated approach to assessing diet quality. The food frequency method is widely used in dietary studies; it describes food intake over the long term better than short-term methods, and it enables ranking respondents to identify those with better and worse diet quality with a relatively low burden on respondents [16,25]. Quantitative dietary methods (e.g., food records, 24 h recall) are more engaging for respondents, especially when records are required for multiple days [25]. Furthermore, prospective dietary methods may also be subject to social desirability bias [62]. Hence, we used a validated descriptive qualitative questionnaire to minimise respondents’ burden and increase participation in the study; despite its limitations, it is a quicker, inexpensive method previously shown to correlate with data obtained from semi-quantitative FFQs [25].

The main limitation is the study’s single-arm, non-randomised design. Randomised, double-blind clinical trials (RCTs) have the highest status in providing scientific evidence. However, RCTs should be preceded by research that identifies the problem and informs the design of the next, more advanced and costly study. In this context, our research fills a gap and provides the first information on the potential use of this simple app.

Some lifestyle data were collected based on respondents’ self-reports, which may have contributed to social desirability bias [62]. Recreational physical activity was assessed using a closed self-report question from the validated KomPAN^®^ questionnaire rather than by an objective or quantitative measure. The question has shown good reproducibility (for interviewer-administered question on recreational physical activity kappa = 0.78) [29,30]. Furthermore, no changes in recreational physical activity levels were observed during the follow-up period (10 weeks or one year), but this does not exclude smaller changes in the duration, frequency or intensity of physical activity that may have contributed to the observed outcomes.

Another limitation relates to the demographic characteristics of the study sample. Approximately 80% of participants were female and about 95% had higher education, indicating an overrepresentation of women and highly educated individuals compared with the general adult population. As a result, the findings may not be generalisable to populations with different sex, educational or socioeconomic distributions. Participants with higher educational attainment may be more interested in health-related topics, more motivated to engage in self-monitoring and more likely to adopt recommended dietary behaviours [63]. Therefore, the observed changes should be interpreted with caution, and future studies should include more diverse and representative populations to confirm the applicability of the findings across broader population groups.

Given the exploratory nature of this first study among app users, we assessed fasting glucose and total cholesterol in capillary blood using a point-of-care device. Although numerical TC values were obtained for most measurements, some results outside the device measurement range were reported only as “high” or “low”, without an exact concentration. This precluded a reliable assessment of changes in the prevalence of elevated TC and limited the statistical analysis to available continuous TC data. In addition, selected cardiometabolic markers had missing values, with a particularly high proportion for TC. Mean imputation may have reduced variability and introduced bias; therefore, findings involving these variables should be interpreted with additional caution. These methodological considerations will be addressed in the next investigation, which will include venous blood sampling and standardised laboratory analyses.

The findings of this study are encouraging and should be further investigated in long-term, prospective, controlled studies that incorporate a larger number of metabolic markers. It would be interesting to investigate changes in app users in more advanced markers of metabolic health, such as detailed blood lipid profiles (high-density lipoproteins, low-density lipoproteins, triglycerides), haemoglobin A1c and insulin levels, as well as inflammation markers (e.g., interleukin-6, C-reactive protein) [54]. Further research could explore the app’s potential for secondary prevention of diet-related diseases. Controlled studies could examine whether the DDApp may support maintenance of weight loss in people with obesity and dietary management in individuals with glucose metabolism disorders or dyslipidaemia. Such studies should determine whether changes in these outcomes differ from those observed under usual care or an appropriate control condition before drawing conclusions about clinical effectiveness or secondary prevention.

## 5. Conclusions

The findings suggest that the Dietary Diary App may serve as a simple, population-oriented self-monitoring tool supporting dietary behaviour change. During the 10-week intervention, favourable within-participant changes were observed in diet quality, body composition and selected cardiometabolic markers. The most consistent dietary finding was a reduction in the non-Healthy Diet Index, indicating lower consumption of less healthy foods. The study also observed short-term improvements in body weight, adiposity measures, blood pressure, fasting blood glucose and total cholesterol. At the one-year follow-up, fewer outcomes remained statistically significant, although lower nHDI, reduced visceral fat tissue and lower systolic blood pressure persisted among voluntary app users.

The findings support the potential usefulness of simplified dietary self-monitoring in promoting healthier eating habits and reducing dietary risk factors associated with non-communicable diseases. However, because this was a single-arm study without a control group, the observed changes cannot be attributed solely to DDApp use. Further long-term, prospective, controlled studies are needed to determine the DDApp’s effectiveness under controlled conditions and whether the observed changes translate into clinically meaningful health benefits.

## Figures and Tables

**Table 1:** Characteristics of the samples at baseline, after 10 weeks and after one year (% of the sample or mean ± SD).

	Invited App Users			Voluntary App Users			*p*-Value for Comparison	
	Baseline	After 10 Weeks	*p*-Value	Baseline	After 10 Weeks	After One Year	Baseline vs. After 10 Weeks	Baseline vs. After One Year
Sample size	62	62	62 vs. 62	30	30	30	30 vs. 30	30 vs. 30
Sample percentage	100	100		100	100	100		
Sex: Female/Male	76/24	76/24	1.000	80/20	80/20	80/20	1.000	1.000
Age (years; mean ± SD)	45.6 ± 11.6	46.0 ± 11.7	0.001	45.0 ± 10.5	45.3 ± 10.5	46.0 ± 10.5	0.016	<0.001
Education			1.000				1.000	1.000
Secondary or lower	5	5		3	3	3		
Higher	95	95		97	97	97		
Residence			0.500				1.000	0.500
Rural	24	21		27	23	20		
Urban	76	79		73	77	80		
Health status compared to peers			0.819				0.607	0.368
Worse	21	21		23	23	23		
The same	55	58		50	57	46		
Better	24	21		27	20	33		
Using a therapeutic diet			0.506				0.160	0.475
No	68	74		60	73	70		
On the doctor’s orders	3	2		3	0	3		
Of one’s own choice	29	24		37	27	27		
Prevalence of chronic diseases ^#^	48	48	1.000	43	43	43	1.000	1.000
Dyslipidaemia	10	10	1.000	10	10	10	1.000	1.000
Glucose abnormalities	18	18	1.000	23	23	27	1.000	1.000
Hypertension	16	16	1.000	10	10	10	1.000	1.000
Other	29	29	1.000	27	27	27	1.000	1.000
Taking medications ^#^	45	45	1.000	40	40	40	1.000	1.000
Cholesterol-lowering drugs	6	6	1.000	3	3	3	1.000	1.000
Glucose-lowering drugs	13	13	1.000	13	13	17	1.000	1.000
Blood pressure-lowering drugs	13	13	1.000	7	7	10	1.000	1.000
Other	29	29	1.000	27	27	27	1.000	1.000
Recreational physical activity			0.475				0.316	0.446
Low	24	18		27	13	27		
Moderate	50	58		47	53	53		
Higher	26	24		27	33	20		
Smoking			0.180				0.083	0.025
Never	60	60		47	47	47		
Past smoking	21	26		27	33	43		
Current smoking	19	15		27	20	10		

Note: SD—standard deviation; ^#^ Respondents could choose multiple answers; *p*-value—significance level (categorical variables: McNemar test, McNemar–Bowker test; continuous variable: Wilcoxon test).

**Table 2:** Invited app users: Means (± SD) of the diet quality and frequency of food consumption at baseline and after 10 weeks.

Variables	Baseline	After 10 Weeks	Change After 10 Weeks		
			AD	RD (%)	*p*-Value
Sample size	62	62			62 vs. 62
Diet quality					
pHDI (% points)	25.8 ± 9.2	28.1 ± 10.0	2.3	8.9	0.052
nHDI (% points)	15.5 ± 6.2	12.1 ± 4.9	−3.4	−21.9	<0.001
pHDI components (times/day)					
Fermented milk drinks	0.38 ± 0.30	0.46 ± 0.37	0.08	21.1	0.023
Fresh cheese curd products	0.38 ± 0.28	0.45 ± 0.35	0.07	18.4	0.155
Vegetables	1.33 ± 0.67	1.57 ± 0.61	0.24	18.0	0.004
Fish	0.12 ± 0.11	0.14 ± 0.13	0.02	16.7	0.265
Fruit	0.95 ± 0.56	1.09 ± 0.66	0.14	14.7	0.088
Pulse-based foods	0.16 ± 0.20	0.18 ± 0.21	0.02	12.5	0.361
Wholemeal bread	0.49 ± 0.43	0.53 ± 0.45	0.04	8.2	0.448
Coarse-ground groats	0.28 ± 0.26	0.29 ± 0.28	0.01	3.6	0.992
White meat	0.42 ± 0.21	0.38 ± 0.22	−0.04	−9.5	0.181
Milk	0.64 ± 0.73	0.53 ± 0.70	−0.11	−17.2	0.199
nHDI components (times/day)					
Energy drinks	0.02 ± 0.07	0.01 ± 0.02	−0.01	−50.0	0.173
White bread	0.80 ± 0.64	0.44 ± 0.40	−0.36	−45.0	<0.001
Tinned meats	0.03 ± 0.09	0.02 ± 0.03	−0.01	−33.3	0.077
Alcoholic beverages	0.10 ± 0.16	0.07 ± 0.11	−0.03	−30.0	0.137
White rice, white pasta, fine-ground groats	0.33 ± 0.22	0.24 ± 0.19	−0.09	−27.3	0.004
Butter	0.83 ± 0.68	0.63 ± 0.53	−0.20	−24.1	0.047
Sweets	0.52 ± 0.43	0.41 ± 0.36	−0.11	−21.2	0.035
Cured meat, smoked sausages, hot dogs	0.53 ± 0.44	0.42 ± 0.33	−0.11	−20.8	0.021
Fried foods	0.35 ± 0.23	0.28 ± 0.24	−0.07	−20.0	0.042
Fast foods	0.07 ± 0.07	0.06 ± 0.07	−0.01	−14.3	0.242
Red meat	0.25 ± 0.23	0.23 ± 0.30	−0.02	−8.0	0.225
Sweetened drinks	0.07 ± 0.25	0.07 ± 0.26	0.00	0.0	0.178
Cheese	0.41 ± 0.26	0.45 ± 0.28	0.04	9.8	0.289
Lard	0.03 ± 0.07	0.05 ± 0.16	0.02	66.7	0.616

Note: SD—standard deviation; pHDI—pro-Healthy Diet Index; nHDI—non-Healthy Diet Index; AD—absolute difference calculated with mean values as follows: AD = after − baseline; RD—relative difference calculated with mean values as follows: RD = [(after − baseline) × 100/baseline]; *p*-value—significance level (Wilcoxon test for paired measurements). Variables were sorted by RD.

**Table 3:** Voluntary app users: Means (± SD) of the diet quality and frequency of food consumption at baseline, after 10 weeks and one year.

Variables	Baseline	After 10 Weeks	Change After 10 Weeks			After One Year	Change After One Year		
			AD	RD (%)	*p*-Value		AD	RD (%)	*p*-Value
Sample size	30	30			30 vs. 30	30			30 vs. 30
Diet quality									
pHDI (% points)	26.0 ± 9.3	27.5 ± 9.6	1.5	5.8	0.358	27.0 ± 9.7	1.0	3.8	0.381
nHDI (% points)	16.7 ± 6.7	12.7 ± 5.8	−4.0	−24.0	0.006	13.5 ± 6.1	−3.2	−19.2	0.017
pHDI components (times/day)									
Pulse-based foods	0.15 ± 0.17	0.20 ± 0.26	0.05	33.3	0.092	0.18 ± 0.26	0.03	20.0	0.679
Fermented milk drinks	0.37 ± 0.29	0.46 ± 0.31	0.09	24.3	0.125	0.41 ± 0.28	0.04	10.8	0.638
Fruit	0.89 ± 0.48	1.05 ± 0.61	0.16	18.0	0.140	0.98 ± 0.64	0.09	10.1	0.478
Vegetables	1.40 ± 0.64	1.63 ± 0.54	0.23	16.4	0.062	1.45 ± 0.62	0.05	3.6	0.674
Fresh cheese curd products	0.42 ± 0.33	0.47 ± 0.38	0.05	11.9	0.535	0.41 ± 0.42	−0.01	−2.4	0.650
Coarse-ground groats	0.30 ± 0.25	0.28 ± 0.27	−0.02	−6.7	0.834	0.25 ± 0.20	−0.05	−16.7	0.609
Wholemeal bread	0.46 ± 0.40	0.42 ± 0.42	−0.04	−8.7	0.609	0.53 ± 0.29	0.07	15.2	0.388
White meat	0.43 ± 0.23	0.38 ± 0.22	−0.05	−11.6	0.203	0.44 ± 0.37	0.01	2.3	0.674
Fish	0.13 ± 0.13	0.11 ± 0.09	−0.02	−15.4	0.638	0.19 ± 0.18	0.06	46.2	0.079
Milk	0.65 ± 0.77	0.48 ± 0.72	−0.17	−26.2	0.158	0.57 ± 0.68	−0.08	−12.3	0.542
nHDI components (times/day)									
Energy drinks	0.02 ± 0.09	0.00 ± 0.02	−0.02	−100.0	0.109	0.02 ± 0.09	0.00	0.0	1.000
White bread	0.91 ± 0.72	0.53 ± 0.49	−0.38	−41.8	0.022	0.62 ± 0.59	−0.29	−31.9	0.046
White rice, white pasta, fine-ground groats	0.35 ± 0.23	0.23 ± 0.19	−0.12	−34.3	0.019	0.25 ± 0.20	−0.10	−28.6	0.049
Tinned meats	0.03 ± 0.04	0.02 ± 0.03	−0.01	−33.3	0.050	0.01 ± 0.03	−0.02	−66.7	0.033
Sweets	0.56 ± 0.45	0.38 ± 0.31	−0.18	−32.1	0.022	0.47 ± 0.45	−0.09	−16.1	0.381
Butter	0.86 ± 0.59	0.60 ± 0.50	−0.26	−30.2	0.076	0.69 ± 0.55	−0.17	−19.8	0.191
Cured meat, smoked sausages, hot dogs	0.61 ± 0.48	0.46 ± 0.40	−0.15	−24.6	0.079	0.44 ± 0.39	−0.17	−27.9	0.020
Alcoholic beverages	0.10 ± 0.14	0.08 ± 0.12	−0.02	−20.0	0.824	0.07 ± 0.12	−0.03	−30.0	0.066
Fried foods	0.38 ± 0.25	0.32 ± 0.25	−0.06	−15.8	0.087	0.34 ± 0.27	−0.04	−10.5	0.286
Red meat	0.30 ± 0.25	0.27 ± 0.38	−0.03	−10.0	0.239	0.30 ± 0.38	0.00	0.0	0.706
Fast foods	0.06 ± 0.04	0.06 ± 0.04	0.00	0.0	0.689	0.07 ± 0.09	0.01	16.7	0.398
Cheese	0.43 ± 0.23	0.48 ± 0.29	0.05	11.6	0.230	0.42 ± 0.27	−0.01	−2.3	0.944
Sweetened drinks	0.03 ± 0.03	0.04 ± 0.09	0.01	33.3	0.554	0.02 ± 0.04	−0.01	−33.3	0.695
Lard	0.04 ± 0.09	0.08 ± 0.21	0.04	100.0	0.944	0.05 ± 0.13	0.01	25.0	0.638

Note: SD—standard deviation; pHDI—pro-Healthy Diet Index; nHDI—non-Healthy Diet Index; AD—absolute difference calculated with mean values as follows: AD = after − baseline; RD—relative difference calculated with mean values as follows: RD = [(after − baseline) × 100/baseline]; *p*-value—significance level (Wilcoxon test for paired measurements). Variables were sorted by RD after 10 weeks.

**Table 4:** Invited app users: Means (±SD) of body composition and cardiometabolic markers at baseline and after 10 weeks.

Variables	Baseline	After 10 Weeks	Change After 10 Weeks		
			AD	RD (%)	*p*-Value
Sample size	62	62			62 vs. 62
Body composition					
Fat mass (kg)	30.5 ± 12.3	28.6 ± 12.4	−1.9	−6.2	<0.001
Fat content (% bw.)	36.6 ± 7.9	35.1 ± 8.5	−1.5	−4.1	<0.001
Body weight (kg)	81.5 ± 18.3	79.7 ± 18.0	−1.8	−2.2	<0.001
BMI (kg/m^2^)	28.6 ± 6.1	28.0 ± 6.1	−0.6	−2.1	<0.001
WHtR (–)	0.54 ± 0.08	0.53 ± 0.08	−0.01	−1.9	0.022
Visceral fat tissue (kg)	2.0 ± 1.6	1.9 ± 1.5	−0.1	−5.0	0.068
Waist circumference (cm)	91.2 ± 14.8	89.7 ± 14.4	−1.5	−1.6	0.019
Skeletal muscle mass (kg)	24.1 ± 6.0	24.0 ± 6.0	−0.1	−0.4	0.646
Fat-free body mass (kg)	51.1 ± 10.4	51.1 ± 10.6	0.0	0.0	0.850
Fat-free mass/Fat mass ratio (–)	1.9 ± 0.9	2.1 ± 0.9	0.2	10.5	<0.001
Muscle mass/Fat mass ratio (–)	0.9 ± 0.5	1.0 ± 0.5	0.1	11.1	<0.001
Cardiometabolic markers					
TC (mg/dL)	199.1 ± 36.6	169.3 ± 25.5	−29.8	−15.0	<0.001
FBG (mg/dL)	108.3 ± 30.1	93.5 ± 17.3	−14.8	−13.7	<0.001
SBP (mmHg)	131.3 ± 16.5	123.5 ± 15.6	−7.8	−5.9	<0.001
DBP (mmHg)	84.8 ± 9.8	80.1 ± 9.3	−4.7	−5.5	<0.001
Continuous interstitial glucose (mg/dL)	106.4 ± 16.9	106.8 ± 11.7	0.4	0.4	0.143
Time in the target glucose range ^#^ (%)	97.4 ± 8.3	98.0 ± 4.2	0.6	0.6	0.779

Note: SD—standard deviation; BMI—body mass index; WHtR—waist-to-height ratio; FBG—fasting blood glucose; ^#^ range of target glucose concentration: 70–180 mg/dL; TC—total cholesterol; SPB—systolic blood pressure; DBP—diastolic blood pressure; AD—absolute difference calculated with mean values as follows: AD = after − baseline; RD—relative difference calculated with mean values as follows: RD = [(after − baseline) × 100/baseline]; *p*-value—significance level (Wilcoxon test for paired measurements); NA—not applicable. Variables were sorted by RD.

**Table 5:** Invited app users: Prevalence (% of the sample) of the body composition and cardiometabolic disorders at baseline (*n* = 62), after 10 weeks (*n* = 62) and one year (*n* = 30).

Variables	Baseline	After 10 Weeks	Change After 10 Weeks		
			AD	RD (%)	*p*-Value
Sample size	62	62			62 vs. 62
Body composition disorders					
Obesity (BMI ≥ 30 kg/m^2^)	31	27	−4	−13	0.500
Waist circumference ≥ 80 [F] or ≥94 cm [M]	76	69	−7	−9	0.344
Obesity (body fat ≥ 30% [F] or ≥25% bw. [M])	87	81	−6	−7	0.125
Central obesity (WHtR ≥ 0.5)	65	65	0	0	1.000
Overweight (BMI = 25–29.9 kg/m^2^)	42	44	2	5	1.000
Cardiometabolic disorders					
High SBP (≥140 mmHg) or DBP (≥90 mmHg)	42	16	−26	−62	<0.001
Elevated FBG (≥100 mg/dL)	58	34	−24	−41	0.001

Note: BMI—body mass index; F—female; M—male; WHtR—waist-to-height ratio; AD—absolute difference calculated with mean values as follows: AD = after − baseline; RD—relative difference calculated with mean values as follows: RD = [(after − baseline) × 100/baseline]; *p*-value—significance level (McNemar test); NA—not applicable. Variables were sorted by RD after 10 weeks.

**Table 6:** Voluntary app users: Means (± SD) of body composition and cardiometabolic markers at baseline, after 10 weeks and one year.

Variables	Baseline	After 10 Weeks	Change After 10 Weeks			After One Year	Change After One Year		
			AD	RD (%)	*p*-Value		AD	RD (%)	*p*-Value
Sample size	30	30			30 vs. 30	30			30 vs. 30
Body composition									
Visceral fat tissue (kg)	1.9 ± 1.8	1.7 ± 1.5	−0.2	−10.5	0.147	1.8 ± 1.7	−0.1	−5.3	0.045
Fat mass (kg)	28.7 ± 9.9	26.5 ± 10.0	−2.2	−7.7	<0.001	27.7 ± 10.1	−1.0	−3.5	0.159
Fat content (% bw.)	36.0 ± 7.4	34.1 ± 7.9	−1.9	−5.3	<0.001	35.4 ± 7.5	−0.6	−1.7	0.153
BMI (kg/m^2^)	27.7 ± 5.3	27.0 ± 5.4	−0.7	−2.5	<0.001	27.2 ± 5.5	−0.5	−1.8	0.068
Body weight (kg)	78.2 ± 17.2	76.3 ± 17.3	−1.9	−2.4	<0.001	76.8 ± 17.2	−1.4	−1.8	0.066
Waist circumference (cm)	89.1 ± 15.8	87.6 ± 15.0	−1.5	−1.7	0.171	87.6 ± 15.6	−1.5	−1.7	0.071
WHtR (–)	0.5 ± 0.1	0.5 ± 0.1	0.0	0.0	0.186	0.52 ± 0.09	0.0	0.0	0.094
Fat-free body mass (kg)	49.5 ± 10.3	49.7 ± 10.8	0.2	0.4	0.262	49.0 ± 10.2	−0.5	−1.0	0.098
Skeletal muscle mass (kg)	23.0 ± 6.0	23.1 ± 6.1	0.1	0.4	0.894	22.7 ± 6.0	−0.3	−1.3	0.077
Fat-free mass/Fat mass ratio (–)	1.9 ± 0.7	2.1 ± 0.8	0.2	10.5	<0.001	2.0 ± 0.7	0.1	5.3	0.289
Muscle mass/Fat mass ratio (–)	0.9 ± 0.3	1.0 ± 0.3	0.1	11.1	<0.001	0.9 ± 0.3	0.0	0.0	0.504
Cardiometabolic markers									
FBG (mg/dL)	111.3 ± 39.3	91.4 ± 18.3	−19.9	−17.9	<0.001	106.0 ± 29.6	−5.3	−4.8	0.069
TC (mg/dL)	201.1 ± 24.8	168.7 ± 21.4	−32.4	−16.1	<0.001	195.3 ± 15.2	−5.8	−2.9	0.202
SBP (mmHg)	129.9 ± 15.9	120.4 ± 16.6	−9.5	−7.3	<0.001	125.0 ± 13.5	−4.9	−3.8	0.015
DBP (mmHg)	84.0 ± 9.3	78.2 ± 9.3	−5.8	−6.9	<0.001	80.8 ± 9.6	−3.2	−3.8	0.107
Continuous interstitial glucose (mg/dL)	106.5 ± 20.2	106.2 ± 13.3	−0.3	−0.3	0.261	104.5 ± 13.4	−2.0	−1.9	0.405
Time in the target glucose range ^#^ (%)	96.4 ± 10.6	97.1 ± 5.5	0.7	0.7	0.241	97.6 ± 3.9	1.2	1.2	0.846

Note: SD—standard deviation; BMI—body mass index; WHtR—waist-to-height ratio; FBG—fasting blood glucose; ^#^ range of target glucose concentration: 70–180 mg/dL; TC—total cholesterol; SPB—systolic blood pressure; DBP—diastolic blood pressure; AD—absolute difference calculated with mean values as follows: AD = after − baseline; RD—relative difference calculated with mean values as follows: RD = [(after − baseline) × 100/baseline]; *p*-value—significance level (Wilcoxon test for paired measurements); NA—not applicable. Variables were sorted by RD after 10 weeks.

**Table 7:** Voluntary app users: Prevalence (% of the sample) of the body composition and cardiometabolic disorders at baseline, after 10 weeks and one year.

Variables	Baseline	After 10 Weeks	Change After 10 Weeks			After One Year	Change After One Year		
			AD	RD (%)	*p*-Value		AD	RD (%)	*p*-Value
Sample size	30	30			30 vs. 30	30			30 vs. 30
Body composition disorders									
Obesity (BMI ≥ 30 kg/m^2^)	33	30	−3	−9	1.000	27	−4	−13	0.500
Waist circumference ≥ 80 [F] or ≥94 cm [M]	63	60	−3	−5	1.000	53	−23	−30	0.250
Obesity (body fat ≥ 30% [F] or ≥25% bw. [M])	80	77	−3	−4	1.000	80	−7	−8	1.000
Overweight (BMI = 25–29.9 kg/m^2^)	30	30	0	0	1.000	30	−12	−29	1.000
Central obesity (WHtR ≥ 0.5)	53	60	7	13	0.625	53	−12	−18	1.000
Cardiometabolic disorders									
High SBP (≥140 mmHg) or DBP (≥90 mmHg)	33	10	−23	−70	0.016	20	−22	−52	0.289
Elevated FBG (≥100 mg/dL)	57	30	−27	−47	0.021	43	−15	−26	0.344

Note: BMI—body mass index; F—female; M—male; WHtR—waist-to-height ratio; AD—absolute difference calculated with mean values as follows: AD = after − baseline; RD—relative difference calculated with mean values as follows: RD = [(after − baseline) × 100/baseline]; *p*-value—significance level (McNemar test); NA—not applicable. Variables were sorted by RD after 10 weeks.

## Data Availability

The data presented in this study are available on request from the corresponding author.

## Supplementary Materials

The following supporting information can be downloaded at https://www.mdpi.com/article/10.3390/nu18183003/s1, Table S1: Characteristics of the sample following the study protocol after 10 weeks and after one year (% of the sample or mean ± SD). Table S2: Voluntary App Users: Assessment of the app usability after one year.

## Abbreviations

The following abbreviations are used in this manuscript:

AD	Absolute Difference
BMI	Body Mass Index
DBP	Diastolic Blood Pressure
DDApp	Dietary Diary App
mHealth	Mobile Health Applications
FBG	Fasting Blood Glucose
FFQ	Food Frequency Questionnaire
ISAK	International Society for Advancement of Kinanthropometry
ITT	Intention to Treat
ITU	Intention to Use
KomPAN^®^	Dietary Habits and Nutrition Beliefs Questionnaire
NCDs	Non-Communicable Diseases
nHDI	non-Healthy Diet Index
pHDI	pro-Healthy Diet Index
PP	Per-Protocol
RD	Relative Difference
SBP	Systolic Blood Pressure
SD	Standard Deviation
TC	Total Cholesterol
WC	Waist Circumference
WHO	World Health Organization
WHtR	Waist-to-Height Ratio
%b.w.	Percentage of body weight
