# Supplementary material for: From Self-Monitoring to Better Health: Changes in Diet Quality, Body Composition and Cardiometabolic Markers Following Use of the Dietary Diary App in a Single-Arm Study
Source: Nutrients. 2026 Sep 14;18(18):3003. doi: 10.3390/nu18183003 (PMC13610070; doi:10.3390/nu18183003)
Supplement: Supplementary file 1 [file nutrients-18-03003-s001.zip › nutrients-4556898-supplementary.pdf]

From Self-Monitoring to Better Health: Changes in Diet Quality, Body Composition and Cardiometabolic Markers Following Use of the Dietary Diary App in a Single-Arm Study

Lidia Wadolowska, Ewa Niedzwiedzka, Beata Stasiewicz, Monika Jablonska, Joanna Kowalkowska

Supplementary materials

Table S1. Characteristics of the sample following the study protocol after 10 weeks and after one year (% of the sample or mean ± SD)

Variables	After 10 weeks[##]	After one year[###]	p-value
Sample size	40	30	40 vs 30
Sample percentage	100	100	
Sex: Female/Male	80/20	80/20	1.000
Age (years; mean ± SD)	46.1 ± 11.2	46.0 ± 10.5	< 0.001
Education			
Secondary or lower	5	3	0.733
Higher	95	97	
Residence			
Rural	27	20	0.469
Urban	73	80	
Health status compared to peers			
Worse	22	23	0.698
The same	53	44	
Better	25	33	
Using a therapeutic diet			
No	73	70	0.508
On the doctor's orders	0	3	
Of one's own choice	27	27	
Prevalence of chronic diseases[#]	50	43	0.580
Dyslipidaemia	10	10	1.000
Glucose abnormalities	20	27	0.511
Hypertension	12	10	0.745
Other	32	27	0.598
Taking medications[#]	48	40	0.532
Cholesterol-lowering drugs	5	3	0.733
Glucose-lowering drugs	12	17	0.622
Blood pressure-lowering drugs	10	10	1.000
Other	32	27	0.598
Recreational physical activity			
Low	17	27	0.583
Moderate	55	53	
Higher	28	20	
Smoking			
Never	58	50	0.353
Past smoking	25	40	
Current smoking	17	10	

Note: SD—standard deviation; [#]Respondents could choose multiple answers; [##]Participants invited in 2026; [###]Voluntary app users who were invited in 2026; p-value—significance level (categorical variables: chi-squared test; continuous variable: Wilcoxon test).

Method of the App Usability Assessment

The frequency of app use during the year was assessed using a question with four possible answers: daily, often, occasionally or never. The System Usability Scale (SUS) developed by Brooke [1] in its Polish version [2] was used as a measure of user-perceived app usability. Answers to positive and negative statements were converted into scores, and the final score for each participant was calculated according to the manual [1,2]. A very good level of app usability was considered when the total SUS score was at least 80 [3-5].

Table S2. Voluntary App Users: Assessment of the app usability after one year.

Variables	After one year
Sample size	30
Sample percentage	100
App assessment with SUS (% of the sample)	
Very good	60
Not very good	40
SUS (mean ± SD)	80.3 ± 14.7

Note: SUS—System Usability Score; Very good—SUS score ≥ 80; Not very good—SUS score < 80; SD—standard deviation.

References

1. Brooke, J. SUS: a "quick and dirty" usability scale. (In:) Jordan, P.W.; Thomas, B.; Weerdmeester, B.A.; McClelland, A.L. (eds.) Usability Evaluation in Industry. Taylor and Francis, London **1996**. Available online: https://www.scirp.org/reference/referencespapers?referenceid=2552035 (accessed on 5 January 2026).
2. Borkowska, A.; Jach, K. Pre-testing of Polish Translation of System Usability Scale (SUS). In: Borzemski, L.; Grzech, A.; Świątek, J.; Wilimowska Z. (eds.) Information Systems Architecture and Technology: Proceedings of 37th International Conference on Information Systems Architecture and Technology – ISAT 2016 – Part I. *Adv. Intell. Syst. Comput.* **2017**, 521, 143–153.
3. SUS calculator. Available online: https://suscalculator.com/?utm_source#calculator (assessed on 15 May 2026).
4. Bangor, A.; Kortum, P.; Miller, J. Determining what individual SUS scores mean: adding an adjective rating scale. *J. Usability Stud.* **2009**, 4, 3, 114–123.
5. Hyzy, M.; Bond, R.; Mulvenna, M.; Bai, L.; Dix, A.; Leigh, S.; Hunt, S. System usability scale benchmarking for digital health apps: meta-analysis. *JMIR Mhealth Uhealth* **2022**, 10, 8, e37290.
